# Staying Current: Developing Just-in-time Evidence-Based Learning Objectives for a Maternal Cardiac Arrest Simulation Curriculum

**DOI:** 10.26502/fccm.92920260

**Published:** 2022-05-23

**Authors:** Andrea D Shields, Jacqueline Battistelli, Laurie Kavanagh, Lara Ouellette, Brook Thomson, Peter Nielsen

**Affiliations:** 1 University of Connecticut Health, Farmington, Connecticut, USA; 2 Baylor College of Medicine, Houston, Texas, USA; 3 University of Texas Health Science Center San Antonio, Texas, USA

**Keywords:** Simulation, Curriculum, Maternal Cardiac Arrest, Learning Objectives, Evidence Process, modified RAND technique, AGREE II Survey

## Abstract

**Background:**

Our objective was to review the latest evidence on resuscitation care for maternal cardiac arrest (MCA) and gain expert consensus on best practices to inform an evidence-based curriculum.

**Methods:**

We convened a multidisciplinary panel of stakeholders in MCA to develop an evidence-based simulation training, Obstetric Life Support™ (OBLS). To inform the learning objectives, we used a novel three-step process to achieve consensus on best practices for maternal resuscitation. First, we reaffirmed the evidence process on an existing MCA guideline using the Appraisal of Guidelines for Research and Evaluation (AGREE II). Next, via systematic review, we evaluated the latest evidence on MCA and identified emerging topics since the publication of the MCA guideline. Finally, we applied a modified Research and Development (RAND) technique to gain consensus on emerging topics to include as additional just-in-time best practices.

**Results:**

The AGREE II survey results demonstrated unanimous consensus on reaffirmation of the 2015 American Heart Association (AHA) MCA guideline for inclusion into the OBLS curriculum. A systematic review with deduplication resulted in 11,871 articles for review. After categorizing and synthesizing the relevant literature, we presented twelve additional best practices to the expert panel using a modified RAND technique. Upon completion, the 2015 AHA statement and nine additional just-in-time best practices were affirmed to inform the OBLS curriculum.

**Conclusions:**

A novel three-step process including reaffirmation of evidence process, systematic review, and a modified RAND technique resulted in unanimous consensus from experts in MCA resuscitation on existing and new just-in-time best practices to inform the learning objectives for an evidence-based curriculum.

## 1. Background

Maternal cardiac arrest (MCA) is a rare and clinically challenging scenario accounting for 1 in 12,000 United States hospital admissions annually, with a pregnancy-related mortality ratio of 17.8 deaths per 100,000 live births in 2009 [1]. By comparison, the pediatric-related mortality ratio is 12.7 deaths per 100,000 (ages 5–14) [2]. While both arrest scenarios are uncommon, cognitive and technical skill mastery for pediatric cardiac arrest is reviewed and tested as a part of Basic Life Support (BLS) and Advanced Cardiac Life Support (ACLS) credentialing. There is no similar credentialing program required or available for pregnancy-related cardiac arrest.

Studies have shown that suboptimal medical response to MCA costs lives. In one study of maternal cardiovascular deaths in Illinois between 2002–2011 [3], 28% of deaths were attributed to preventable causes, including those related to healthcare response and mismanagement. In a statewide review of maternal mortality performed in California between 2002 and 2006, the California Pregnancy Associated Mortality Review determined that 23% of cardiovascular-associated maternal deaths were preventable, with provider factors contributing to 68.8% of deaths [4]. This paradigm has created a knowledge gap across specialties, and even at the highest levels of care, as demonstrated in a 2000–2002 study of residents & faculty [5]. Respondents, including representatives from anesthesia, obstetrics (OB), and emergency medicine departments, were asked how to resuscitate pregnant women properly. The study revealed that the knowledge of even highly trained specialists was “variable and often inadequate,” with only 15% earning a score that would be considered passing based on ACLS course standards [5]. Additionally, other studies [6–9] note that a knowledge gap exists even for OB specialists trained in ACLS. These facts suggest a severe deficiency in the existing standards for treatment of MCA.

In 2015 the American Heart Association (AHA) published the *Scientific Statement on Cardiac Arrest in Pregnancy*,[1] highlighting the need for healthcare professionals to employ specialized interventions when resuscitating a pregnant woman and calling for the development of a standardized training course. In addition, in 2012 the Society for Obstetric Anesthesia and Perinatology (SOAP) board of directors issued a consensus statement calling for improving maternal resuscitation by providing healthcare providers education and operational strategies, emphasizing communication/behavior, latent system errors, and performance testing [10]. Despite the availability of accepted, evidence-based practices for MCA response [1,10,11], the medical training sector in the U.S. lacks a standardized approach for its management and immediate post-arrest care.

A standardized, evidence-based training curriculum for MCA response is urgently required. To address this need, a team of stakeholders are currently developing a simulation-based training package for in-hospital and out-of-hospital MCA named Obstetric Life Support™ (OBLS). This article describes an innovative three-step approach used to identify and achieve consensus on current best practices to train resuscitation skills needed during MCA.

## 2. Methods

After being awarded a grant from the National Institutes of Health (AHRQ FOA PA16–420) and gaining Baylor College of Medicine Institutional Review Board (IRB) approval (#H-48730), a novel validation process was used to create just-in-time learning objectives to inform the creation of a new hybrid simulation curriculum to train emergency medicine services (EMS) providers and in-hospital personnel to manage maternal cardiac arrest (MCA) called OBLS. During the initial phase, an expert panel was formed, comprised of researchers and national stakeholders in MCA. The researchers identified stakeholders in pre-hospital and in-hospital MCA across North America (Additional File 1) by online searches, reviewing authors for peer-reviewed manuscripts and guidelines, and contacting national organizations and asking to be referred to relevant staff. These stakeholders formed the expert panel and agreed to develop the OBLS simulation curriculum to train EMS providers and in-hospital personnel to manage MCA.

### Step 1: Reaffirmation of the 2015 AHA Statement of MCA in Pregnancy

The OBLS expert panel was asked to apply an AGREE II assessment tool to the 2015 AHA guidelines on MCA. The AGREE II tool contains 23 items organized into six quality domains that assess the methodological quality and applicability of guideline recommendations. The six domains are: scope and purpose; stakeholder involvement; rigor of development; clarity of presentation; applicability; and editorial independence. Using a seven-point scale (1 = strongly disagree to 7 = strongly agree) appraisers independently reviewed and assessed the validity and usefulness of the 2015 AHA Statement.

### Step 2: Systematic Review of the Literature

A trained medical librarian developed two comprehensive literature searches using the Medline and OVIDSP databases. Medical Subject Headings (MeSH) were utilized, and keywords and synonyms (See Additional Files 3). Given that the AHA guidelines were published in 2015, the researchers limited the search to 2014 -November 2018. The researchers approved the terms, and the librarian developed a second search strategy using the same publication date range. The search was then translated to two additional databases: Embase and The Cochrane Library. Final searches were run on November 29, 2018. In February 2019, the researchers decided to include the CINAHL Plus with Full Text database into the initial search to ensure the search captured all relevant articles. The researchers updated the search results from the first search initially run in November 2018. The search strategy was translated to the CINAHL database on February 7, 2019. The CINAHL search totals were combined with the initial search results from the searches (Medline OVID, Embase, and the Cochrane Library) run on November 29, 2018. A deduplication process was performed for the combined totals. The updates of the initial searches were also run on Medline OVID, Embase, and the Cochrane Library on February 7, 2019. The updates from the initial searches were combined and underwent the deduplication process on February 11, 2019. The updated search totals were added to the CINAHL search results and deduplicated again. The total 2,249 results of the updates and the CINAHL searches were then delivered to the researchers for review and to undergo the inclusion and exclusion process. The search strategies are available in Additional File 4.

The researchers reviewed the articles for relevance to the project. After categorizing and determining relevance, the researchers developed key questions, abstracted, and graded the literature, and defined inclusion and exclusion criteria. Four independent reviewers selected studies to be included based on consensus on whether they met the inclusion and exclusion criteria. An independent reviewer was available if the researchers could not reach agreement but was not utilized. A synthesis of the relevant literature for each key question was performed focusing on validity and reliability of admissible evidence at the individual study level. The researchers developed overall summary statements regarding specific tasks for managing MCA and extracted literature into a support summary table for each key question.

### Step 3: Modified RAND Technique to Update the AHA statement

These overall summary statements were presented to the expert panel (Table 1) using a modified RAND technique. Via REDCap, a secure web platform for surveys, the researchers invited 24 members of the expert panel to evaluate and rank the statements using a Likert scale (Table 2). Expert panel members who had expertise in areas other than obstetrics, anesthesiology, emergency care or resuscitation were not included. An *a priori* determination of consensus to support the summary statements included an average score of 5.0 or greater; scores <5.0 were automatically reviewed in round two. The researchers found new level A evidence supporting the AHA’s 2015 statement “We recommend against the routine prehospital cooling of patients after return of spontaneous circulation (ROSC) with rapid infusion of cold intravenous fluids.” Therefore, the researchers asked the expert panel to reaffirm the statement as written. After completing the first round, an average ranking score for each statement was calculated and comments for modifications to the statement were incorporated into the consensus statements.

The second round consisted of a face-to-face meeting of the expert panel on January 31 – February 1, 2019, with a consensus discussion for each revised statement led by the researchers. The moderated discussion involved presentation of the individual statements and summary of evidence to support the update. This was followed by an approximately 20-minute discussion, with more time given for a statement if needed. The discussion focused on statement content and wording. Using Poll Everywhere, a live and anonymous audience participation software, the researchers surveyed the expert panel again using the same Likert scale. Stakeholder also answered, “Should this statement be incorporated into the OBLS curriculum?” To be considered for affirmation and inclusion into the curriculum, an *a priori* result of 4.0 or greater, and >80% consensus was set. After the face-to-face meeting, the statements were evaluated based on ranking and consensus around inclusion into the curriculum, and the researchers further refined the statements. No further rounds were performed due to significant agreement around statements. The final statements that met criteria were then sent to the expert panel for affirmation.

## 3. Results

### Step 1: Reaffirmation of the 2015 AHA Statement of Managing Cardiac Arrest in Pregnancy

Sixteen expert panelists representing expertise in resuscitation, obstetrics, anesthesiology, and emergency care were invited, and fourteen members responded to appraise the 2015 AHA *Statement of Managing Cardiac Arrest in Pregnancy* via the AGREE II tool (Additional File 2). Scores for the domains ranged from a low of 58% (Domain 2. Stakeholder Involvement) indicating that the stakeholders did not think that the guidelines satisfied the criteria for this domain to a high of 90% (Clarity of Presentation) indicating the guidelines mostly satisfied the criteria for this domain. Scores were >80% for four of the six domains: Scope and Purpose (82%), Rigor of Development (70%), Clarity of Presentation (90%), Editorial Independence (85%) and for the Overall Assessment (75%). There was unanimous consensus to recommend the guideline for use in the OBLS curriculum, with eight panelists responding “Yes” and six responding “Yes with modifications.” A qualitative synthesis of the recommended modifications is listed in Table 3.

### Step 2: Systematic Review of the Literature

Following both searches and the second deduplication process, the results totaled 9,622 records (Figure 1). An additional 296 records from other sources were combined with this search and deduplicated resulting in 7,152 full-text articles for review. Three reviewers (A.S., J.V. and L.C.) screened these 7,152 articles independently and in duplicate, using the same inclusion and exclusion criteria from the search strategy. A total of 5,449 articles were excluded because they did not meet focus population, intervention, or outcome. Four reviewers (A.S., J.B., P.N., and B.T.) abstracted 1,703 full-text articles, looking at key questions to appraise their quality. A total of 375 studies were included in the qualitative synthesis and 112 studies for quantitative synthesis. From this synthesis, the researchers developed 12 statements for expert panel consideration.

### Step 3: Modified RAND Technique to Update the AHA statement

During the first round, 23 expert panel members were surveyed on the updated AHA statements. Descriptive statistics were performed (Table 4). After the first round, the experts all agreed to reaffirm the AHA 2015 statement: “We recommend against the routine pre-hospital cooling of patients after ROSC with rapid infusion of cold intravenous fluids.” There was also a very high level of consensus (“5” or greater) on only one summary statement: “Training emergency room physicians in perimortem cesarean delivery (PMCD) is recommended so that PMCD can be immediately performed upon arrival to the hospital for out-of-hospital maternal cardiac arrest (MCA) without ROSC.” The remainder of the statements had a “high” or “moderate” level of consensus but did not meet the *a priori* specified rank of “5” or greater to be considered for affirmation. Following the second round, 8 of the 11 statements met the *a priori* rank of “4” of greater for affirmation. Two statements were voted on as dangerous/inappropriate: “First responders should initiate and maintain bag-mask valve (BMV) techniques until arrival at a hospital with a more experienced laryngoscopist” and “EMS should deploy highly specialized paramedics in addition to regular EMS crew in cases of suspected MCA” to less important: “The use of a ketamine-based anesthesia package should be considered for patients with ROSC who have undergone PMCD in settings without immediate anesthesia availability”. These three statements were removed from further consideration as they all received a lower score following the face-to-face discussion compared to the first round. Because there was consensus for all remaining statements, further rounds were not performed. The team refined the remaining nine statements and sent them to the expert panel. All nine statements were affirmed for inclusion into the curriculum (Table 5).

## 4. Discussion

National OB training platforms that are currently employed by specialty organizations dedicate very small portions of their courses, if any, to teaching how to manage MCA. The programs that do exist are narrowly focused on OB and family practice physicians and nurses, limiting broad application to other specialists, and emergency medical technicians. Additionally, they do not require summative MCA-simulation-testing as a course completion requirement. Evidence suggests, however, that continually improving cardiac-response processes (i.e., through simulation training) manifests in improved patient outcomes. Andersen et al. published an article in the Journal of the American Medical Association (JAMA) in 2016 [12] which evaluated whether hospital *process composite performance measures* of in-hospital cardiac arrest care quality are associated with patient outcomes. The authors state: “After adjustment, each 10% increase in a hospital’s process composite performance was associated with 22% higher odds of survival (adjusted odds ratio, 1.22; 95% CI, 1.08–1.37; P = .01). Hospital process composite quality performance was also associated with favorable neurologic status at discharge (P = .004)”[12], demonstrating that standardized processes for treating in-hospital cardiac arrest improve patient outcomes. It is reasonable to expect a similar result as standards for *maternal* cardiac arrest simulation training (and subsequently, hospital-related processes) are instituted throughout the medical sector.

The OBLS expert panel utilized a novel three-step process to reaffirm the 2015 scientific statement, review the evidence to update the 2015 AHA guidelines, and achieve consensus around the proposed statements to arrive at just-in-time best practices to supplement the 2015 AHA scientific statement on managing cardiac arrest in pregnancy. The Agency for Healthcare and Research Quality U.S. Preventive Services Task Force Procedure Manual has previously described the reaffirmation evidence process of clinical practice guidelines [13]. An AGREE II assessment of the 2015 AHA guidelines on Maternal Cardiac Arrest was used to assess the quality of the guidelines, a critical step to evaluate the quality of the guideline used to inform the development of the OBLS curriculum. Overall, the most significant limitation appeared to be a lack of stakeholder involvement and applicability. Reviewers noted that the AHA does not typically include the target population’s voice. However, given the ethical and emotional issues involved in such a complex topic, their perspective would have been an important addition. As for applicability, reviewers noted that the guideline lacked implementation guidance, information related to cost, and specific criteria to develop an auditing program.

With these limitations in mind, the second step involved performing a systematic review limited to the evidence since the 2015 AHA guidelines on Cardiac Arrest in Pregnancy. Prior systematic reviews in this topic area are notable for the lack of level A evidence [1, 11]. In light of this, our review was not limited to experimental designs, resulting in a large volume of new information cardiac arrest management in pregnancy for qualitative and quantitative synthesis. While the expert panel did not adopt all the proposed summary statements for the OBLS curriculum, they affirmed or reaffirmed nine topic areas for inclusion, resulting in substantial modifications to the current guidelines around terminology, operators for resuscitative delivery, point-of-care ultrasound, and extracorporeal life support. In addition, many of these topic areas addressed the limitations of the 2015 AHA guideline highlighted by the expert panel during the Agree II assessment.

The OBLS expert panel then utilized a modified RAND technique to arrive at consensus on the summary of evidence for the 2015 AHA guidelines. Nominal consensus technique such as the RAND technique have been previously described [14, 15]. Because the face-to-face discussion involved a larger group than has been previously described with a RAND technique, and because we performed a follow-up round after the face-to-face discussion to affirm the evidence, we considered our process to be a modification of the RAND technique.

Our results demonstrated enthusiastic affirmation of several updates to the 2015 AHA statement, consistent with the current 2–5-year timeline for updating guidelines described in the literature [16–18]. Additionally, this process excluded three summary statements after the face-to-face discussion at the Expert Panel meeting. This highlighted the importance of exchanging ideas between the expert panel members, resulting in a more accurate understanding of pre and in-hospital practices and the potential positive or negative impact these recommendations may have on practice.

Limitations of our methodology included the inability to calculate analytic statistics of individual responses during the consensus rounds. Fortunately, the *a priori* definition of consensus was readily achieved by the group after three rounds, and therefore statistical analysis of individual responses to determine further rounds was unnecessary. Another limitation was that experts had to self-select “0” if they did not feel qualified to rank a statement. This may have resulted in some experts answering questions that they were not fully qualified to answer. However, the high degree of consensus following the third round suggests this self-selection was not likely to significantly bias the consensus process.

Despite these limitations, this novel process was systematic and thorough and brought together a multidisciplinary panel of experts from all regions of North America, including both pre and in-hospital settings. We believe this is the most diverse group of experts convened around this topic area which permitted a robust discussion on the newest evidence to update the 2015 AHA scientific statement. Existing and new just-in-time best practices will inform the learning objectives for an evidence-based simulation training package relevant for a wide range of medical disciplines to improve the care of maternal cardiac arrest.

## Supplementary Material

Additional file

## List of abbreviations

MCA: maternal cardiac arrest
OBLS: Obstetric Life Support™
AGREE II: Appraisal of Guidelines for Research and Evaluation
RAND: Research and Development
AHA: American Heart Association
ACLS: Advanced Cardiac Life Support
SOAP: Society for Obstetric Anesthesia and Perinatology
EMS: Emergency Medical Services
MeSH: Medical Subject Headings
ROSC: Return of spontaneous circulation
PMCD: Perimortem cesarean delivery
BMV: Bag-Mask-Valve
JAMA: Journal of the American Medical Association
