# Supplementary material for: Staying Current: Developing Just-in-time Evidence-Based Learning Objectives for a Maternal Cardiac Arrest Simulation Curriculum
Source: Cardiol Cardiovasc Med. Author manuscript; Available in PMC 2022 Jul 19. (PMC9296142; doi:10.26502/fccm.92920260)
Supplement: Additional file [file NIHMS1810053-supplement-Additional_file.docx]

| 1. Training emergency room physicians in PMCD is recommended so that PMCD can be immediately performed upon arrival to the hospital for out-of-hospital MCA without ROSC  (Class I; Level of Evidence C). |
| --- |
| 2. PMCD should be immediately performed in a pregnant patient with a fundus height at or above the umbilicus with a non-shockable rhythm (versus proceeding with standard ACLS then PMCD after 4 minutes as would be recommended in pregnant patients with a shockable rhythm)  (Class I; Level of Evidence C). |
| 3. The term "perimortem cesarean delivery" should be replaced with the term "resuscitative hysterotomy" to more correctly describe the purpose/indication and increase the sense of urgency for performing this procedure. |
| 4. First responders should initiate and maintain BMV techniques until arrival at a hospital with a more experienced laryngoscopist. |
| 5. EMS should deploy highly specialized paramedics in addition to regular EMS crew in cases of suspected MCA. |
| 6. The use of a ketamine-based anesthesia package should be considered for patients with ROSC who have undergone PMCD in settings without immediate anesthesia availability  (Class IIb, Level of Evidence C). |
| 7. The use of extracorporeal life support (ELS, or eCPR) should be strongly considered for management of maternal cardiac arrest complicated by refractory cardiopulmonary resuscitation (CPR) in an ECMO center with capacity to care for critically ill pregnant patients (Class IIa; Level of Evidence C). |
| 8. The use of ELS or eCPR should be considered for organ procurement in pregnant patients post- arrest with circulatory determination of death (Class llb; Level of Evidence C). |
| 9. Where available, POC-US should be used in the management of MCA for identification of an intrauterine pregnancy and quick determination of gestational age to guide decision making on PMCD (Class IIa; Level of Evidence C). |
| 10. POC-US should be considered for use during MCA in emergency protocols for identification of potentially reversible causes of cardiac arrest, identification of cardiac contractility activity without palpable pulse for clinical reclassification of pulseless electrical activity, and identification of the absence of cardiac contractility where further attempts at resuscitation may be unsuccessful (Class IIa; Level of Evidence C).* *POC-US should not interfere with CPR, thus should only be performed during brief pauses in CPR. |
| 11. The use of POC-US by prehospital providers for diagnosis and management of maternal cardiac arrest should only be utilized in research protocols (Class IIa, Level of Evidence C). |
| 12. We recommend AGAINST the routine prehospital cooling of pregnant patients after ROSC with rapid infusion of cold intravenous fluids (Class III: No Benefit; Level of Evidence A). |

**Table 1:** First Round Summary Statements Reviewed by Expert Panel.

| **Ordinal Scale** | **Ranking** | **Description** |
| --- | --- | --- |
| 0 | No ranking | I do not have sufficient information OR I am not an expert in this area and therefore cannot make a determination on the ranking. |
| 1 | Dangerous/Inappropriate | This recommendation is inappropriate and is actually dangerous to the patient or other health care providers. This recommendation should be removed from consideration for incorporation into the guideline. |
| 2 | Not important/Remove from consideration | This recommendation is inconsequential or of so little importance that it should be removed from consideration for incorporation into the guideline. |
| 3 | Less important | This recommendation is the lowest priority recommendation. It should only be considered for incorporation into the guideline with further discussion and consensus. |
| 4 | Average importance | This recommendation is moderately important. It should only be considered for incorporation into the guideline with further discussion and consensus. |
| 5 | More important | This recommendation is important and should be considered for incorporation into the guideline. |
| 6 | Extremely important | This is a critical, life-saving recommendation. Without incorporation of this recommendation into the current guidelines, the life of the mother may be lost. |

**Table 2:** First Round Likert Scale to Rank Summary Statements

| Could have definitely benefited from more stakeholders among patients, organizations |
| --- |
| There are out of hospital considerations which are not clearly addressed for MCA |
| Due to the lack of clear evidence this statement represents the most current knowledge and expert consensus on management of MCA |
| Outdated areas include the use of vasopressin and information related to post arrest hypothermia |
| The section on EMS care should be further developed |
| More specific direction on PMCD should be included (including potential operators) |

**Table 3:** Suggested Modifications to AHA Guidelines by Expert panel

| **Statements** | **Pre Mean** | **Post Mean** | **Pre SD** | **Post SD** |  |
| --- | --- | --- | --- | --- | --- |
| Training emergency room physicians in perimortem cesarean delivery (PMCD) is recommended so that PMCD can be immediately performed upon arrival to the hospital for out-of-hospital maternal cardiac arrest (MCA) without return of spontaneous circulation (ROSC). | 5.17 | 5.74 | 1.03 | 0.45 |  |
| PMCD should be immediately performed in a pregnant patient with a fundus height at or above the umbilicus with a non-shockable rhythm (versus proceeding with standard ACLS then PMCD after 4 minutes as would be recommended in pregnant patients with a shockable rhythm). | 4.78 | 5.35 | 1.13 | 0.80 |  |
| The term "perimortem cesarean delivery" should be replaced with the term "resuscitative hysterotomy" to more correctly describe the purpose/indication and increase the sense of urgency for performing this procedure. | 4.87 | 5.18 | 0.97 | 1.39 |  |
| First responders should initiate and maintain BMV techniques until arrival at a hospital with a more experienced laryngoscopist arrives | 4.57 | 1.92 | 1.59 | 1.29 |  |
| EMS should deploy highly specialized paramedics in addition to regular EMS crew in cases of suspected MCA. | 4.87 | 2.52 | 1.32 | 0.98 |  |
| The use of a ketamine-based anesthesia package should be considered for patients with ROSC who have undergone PMCD in settings without immediate anesthesia availability. | 4.22 | 3.56 | 1.17 | 1.45 |  |
| The use of extracorporeal life support (ELS, or eCPR) should be strongly considered for management of maternal cardiac arrest complicated by refractory cardiopulmonary resuscitation (CPR) in an ECMO center with capacity to care for critically ill pregnant patients. | 4.86 | 5.40 | 0.94 | 0.76 |  |
| The use of ELS or eCPR should be considered for organ procurement in pregnant patients post-arrest with circulatory determination of death. | 4.33 | 4.53 | 0.97 | 0.77 |  |
| Where available, POC-US should be used in the management of MCA for identification of an intrauterine pregnancy and quick determination of gestational age to guide decision making on PMCD. | 4.26 | 4.96 | 1.29 | 0.75 |  |
| POC-US should be considered for use during MCA in emergency protocols for identification of potentially reversible causes of cardiac arrest, identification of cardiac contractility activity without palpable pulse for clinical reclassification of pulseless electrical activity, and identification of the absence of cardiac contractility where further attempts at resuscitation may be unsuccessful. | 4.90 | 4.78 | 0.89 | 0.85 |  |
| The use of POC-US by prehospital providers for diagnosis and management of maternal cardiac arrest should only be utilized in research protocols. | 3.87 | 4.30 | 1.46 | 1.02 |  |
| Legend. Pre = ranking + SD after first round, prior to Expert Panel meeting, post = ranking +SD after second face-to-face consensus round at Expert Panel Meeting. | | | | | |

**Table 4:** Rankings and Standard Deviations for Statements before and after Expert Panel Meeting using the modified RAND consensus process.

**1. Use 'resuscitative cesarean delivery' (RCD) instead of 'perimortem cesarean delivery.'**

2. Providers staffing emergency departments should be trained in resuscitative cesarean delivery (RCD).

3. Perform resuscitative cesarean delivery (RCD) immediately in a pregnant patient with a fundus height at or above the umbilicus with a non-shockable rhythm.

4. The use of extracorporeal membrane oxygenation (ECMO, or eCPR) may be considered for the management of maternal cardiac arrest when there is no return of spontaneous circulation (ROSC).

5. The use of extracorporeal life support (ELS or eCPR) should be considered for organ procurement in pregnant patients post-arrest after circulatory determination of death.

6. Where available and when pregnancy stage and gestational age is uncertain, point of care ultrasound (POC-US) may be used in the management of maternal cardiac arrest (MCA) for identification of an intrauterine pregnancy and quick determination of gestational age to guide decision making on resuscitative cesarean delivery (RCD).

7. In maternal cardiac arrest (MCA) with return of spontaneous circulation (ROSC), consider using point of care ultrasound (POC-US) in emergency protocols for identification of potentially reversible causes of cardiac arrest.

8. Where properly trained and resources available, prehospital providers may use point of care ultrasound (POC-US) for diagnosis and management of maternal cardiac arrest (MCA).

**Table 5:** Third Round. Final Statements Affirmed for OBLS Curriculum


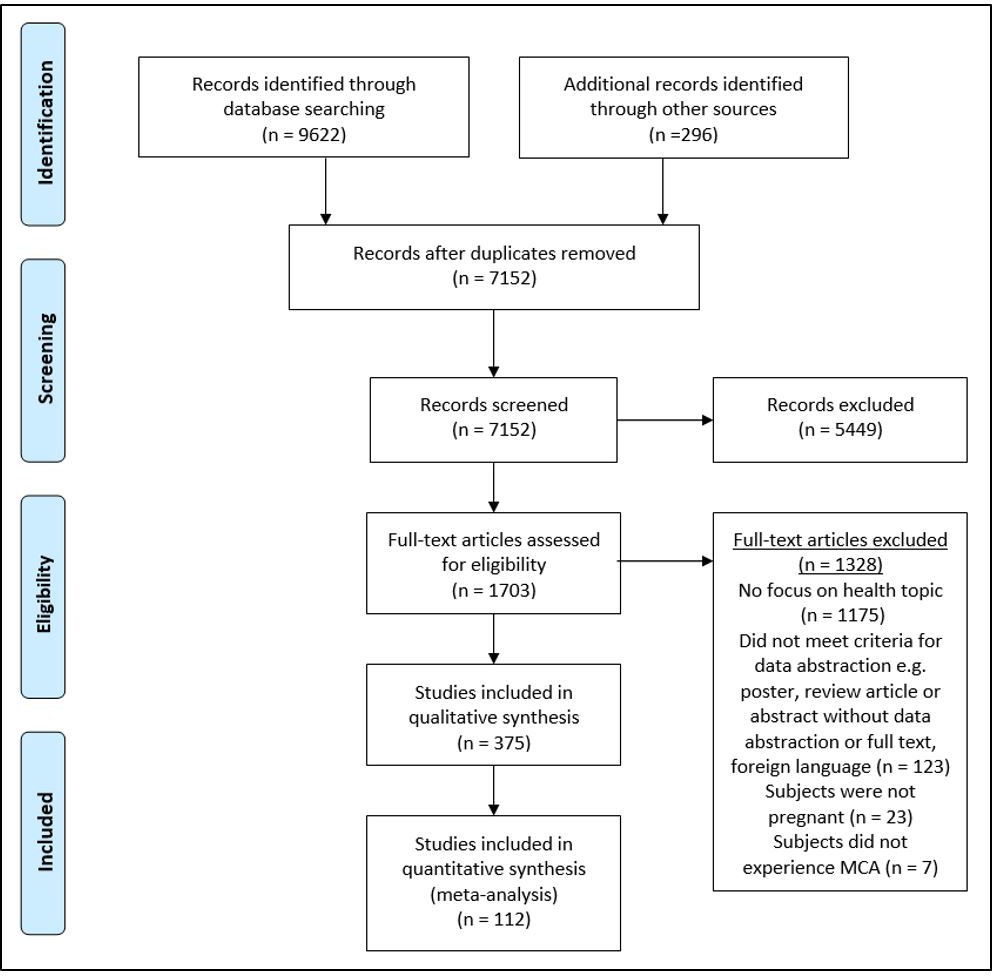


**Figure 1:** Systematic Review Flowchart

Additional Files.

Title: File 1. OBLS expert panel disclosures

Title: File 2. OBLS expert panel scores for the Agree II assessment

Title: File 3: MeSH search terms

Title: File 4. Systematic review methodology

File 1. OBLS expert panel members and disclosures.

| Disclosures | | | | | | | | |
| --- | --- | --- | --- | --- | --- | --- | --- | --- |
| Expert Panel Member | Employment | Research Grant | Other Research Support | Speaker's Bureau | Expert Witness | Ownership Interests | Consultant/ Advisory Board | Other |
| Benjamin Abella | Resuscitation Science at the University of Pennsylvania | Med- tronic Found- ation, Physio- Control Inc., PCORI, American Heart Assoc- iation, NIH NHLBI | None | CR Bard, Physio- Control Inc. | None | None | Neuro- prote Xeon, Inc | None |
| Elumalai Appachi | Baylor College of Medicine, Children’s Hospital of San Antonio | None | None | None | None | None | None | None |
| Julie Arafeh | Stanford University | None | None | None |  | None | None | None |
| Les R. Becker | MedStar Health | None | None | None | None | None | None | None |
| Shad Deering | Children’s Hospital of San Antonio | None | None | None | None | None | None | None |
| Mary Ann Faucher | Baylor University | None | None | None | None | None | None | None |
| Afshan Hameed | University of California Irvine | None | None | None | None | None | None | None |
| Heidi King | Defense Health Agency | None | None | None | None | None | None | None |
| Miranda Klassen | Amniotic Fluid Embolism Foundation | None | None | None | None | None | None | None |
| Monica Lutgendorf | U.S. Navy | None | None | None | None | None | None | None |
| Lissa Magloire | Mednax, Texas Perinatal Group | None | None | None | None | None | None | None |
| Charles Minard | Baylor College of Medicine | None | None | None | None | None | None | None |
| Vincent Mosesso | UPMC/University of Pittsburgh | None | None | None | Occas- ional cases, none related to this issue | None | CARES Over- sight Board; Medical Director Sudden Cardiac Arrest Association | Medical Director, Advanced Medical Life Support Program, NAEMT |
| Jeffrey Quinlan | United State Navy | None | None | None | None | None | None | None |
| DeWayne Pursley | Harvard Medical Faculty Physicians | None | None | None | None | None | National Institute for Child Health/Human Develop-ment | None |
| Carl Rose | Mayo Clinic | None | None | None | None | None | None | None |
| Cheryl Roth | Honor Health | None | None | None | None | None | None | None |
| James Ruiter | Salus Global | None | None | None | None | None | None | None |
| Brian Schaeffer | City of Spokane Washington | None | None | None | None | None | None | None |
| Amir Shamshirsaz | Baylor College of Medicine | None | None | None | None | None | None | None |
| Fernando Stein | Baylor College of Medicine | None | None | None | None | None | None | None |
| Paloma Toledo | Northwestern University/Feinberg School of Medicine | None | None | None | None | None | None | None |
| Rebecca Cypher | Cypher Maternal-Fetal Solutions | None | None | None | None | None | None | None |
| Sondie Epley | University Hospital | None | None | None | None | None | None | None |
| Stephen Rahm | Centre for Emergency Health Sciences Healthcare Innovation and Sciences Centre | None | None | None | None | None | None | None |
| Stephanie Stokes | CHRISTUS Health | None | None | None | None | None | None | None |
|  |  |  |  |  |  |  |  |  |
| Staff |  |  |  |  |  |  |  |  |
| Jacqueline Battistelli | Baylor College of Medicine | NIH OBLS | None | None | None | None | None | None |
| Laurie Kavanagh | Baylor College of Medicine | NIH OBLS | None | None | None | None | None | None |
| Peter Nielsen | Baylor College of Medicine | NIH OBLS | None | None | None | None | None | None |
| Andrea Shields | Baylor College of Medicine | NIH OBLS | None | None | None | None | None | None |
| Brook Thomson | Baylor College of Medicine | NIH OBLS | None | None | None | None | Society of OB/GYN Hospitalists Board | ACOG ECO Faculty/Development Panel, ACOG Simulation Work Group |
|  |  |  |  |  |  |  |  |  |

This table represents the relationships of OBLS expert panel, working group and staff members that may be perceived as actual or reasonably perceived conflicts of interest as reported on the Disclosure Questionnaire administered, which all members of the writing group were required to complete and submit between by January 2019. A relationship is “significant” if (a) the person receives $10000 or more during any 12-month period, or 5% or more of the person’s gross income; or (b) the person owns 5% or more of the voting stock or share of the entity or owns $10000 or more of the fair market value of the entity. A relationship is “modest” if it is less than “significant” under the preceding definition

File 2. OBLS Expert Panel Scores for each Domain of the Agree II Assessment and Overall Approval of the 2015 AHA Statement of Managing Cardiac Arrest in Pregnancy.

| Domain | Focus | Score |
| --- | --- | --- |
| Domain 1:  Scope  and Purpose | Evaluates the overall aim, specific health question,  and target population of the guideline. | 82% |
| Domain 2:  Stakeholder Involvement | Evaluates the degree to which the appropriate stakeholders  developed the guidelines and represents the views and  preferences of the target population | 58% |
| Domain 3:  Rigor  of  Development | Relates to the process used to gather and synthesize evidence including grading and summarizing, as well as the methods to formulate recommendations and to update them | 72% |
| Domain 4:  Clarity  Of Presentation | Evaluates the presentation and format of the guidelines including language, structure, and format | 90% |
| Domain 5:  Applicability | Evaluates the consideration of likely facilitators or barriers to implementation, strategies to address them,  and resources needed to apply the guidelines | 65% |
| Domain 6:  Editorial  Independence | Is concerned with the guidelines being formulated  without competing interests | 85% |
| Overall  Assessment | Rating of the overall quality of the guideline and whether the guideline would be recommended for use in practice | 75% |
| Approve | Without modifications | 57% |
|  | With modifications | 43% |
|  | Total | 100% |

File 3: MeSH search terms

ACLS, ACLS drugs, Advanced life support in pregnancy or ACLS guidelines, amniotic fluid embolism, AFE, airway/management, amniotic fluid embolism, anaphylactoid embolism, anaphylactoid reaction, anesthesia in obstetrics, aortocaval compression, aspiration risk in pregnancy/cardiac arrest, automated external defibrillator, best practices cardiac arrest, cardiac arrest, cardiac death, cardiac disease, cardiopulmonary arrest (CPR), cardiovascular changes, cardiovascular drugs, chest compressions, compressions, CPR and large breasts, CPR and obesity, CPR pregnancy guidelines, critical care in pregnancy/obstetrics, defibrillation, e-health, e-learning, extracorporeal membrane oxygenation (ECMO), etiologies of cardiac arrest in pregnancy, heart attack, hypertensive emergency in pregnancy, IV access in pregnancy/obstetrics, left uterine displacement, manikin, mannequin, myocardial infarction, out of hospital cardiac arrest in pregnancy, perimortem cesarean section, physiologic changes in cardiac arrest in pregnancy, physiologic changes of pregnancy, post- ROSC treatment in pregnancy/postpartum, post-arrest, post-ROSC outcomes in pregnancy/postpartum, postmortem cesarean, pre-arrest, racial disparities in cardiac arrest: clinical implications, pulmonary embolism in pregnancy, rapid sequence intubation, rescue breaths, resuscitation, resuscitative hysterotomy, sepsis in pregnancy, simulation (low and high fidelity), and stroke in pregnancy and virtual reality.

File 4. Systematic Review Methodology

Two comprehensive literature searches were developed by a trained medical librarian for a search using the Medline and OVIDSP database. The first search was to find as many relevant articles as possible and information on “Heart Arrest During Pregnancy.” Three encompassing concepts were explored and detailed, that of “Pregnancy,” “Heart Arrest,” and “Resuscitation.” The concepts were then combined to encapsulate the most applicable materials. Medical Subject Headings (MeSH) were utilized, as well as keywords and synonyms. Related terminology was also pulled from relevant articles. These terms were then searched using the title, abstract, and keyword fields within the OVID MEDLINE database. Once the search strategy was complete, it was submitted for approval and final editing. For the initial search, the researchers decided to only search between 2014 to current (November 2018) given that the AHA guidelines were published in 2015. The Publication Data range was limited to items from the year 2000 to current.  Once approved by the researchers, a second search strategy was developed. The second strategy focused on the educational and training aspects of “Heart Arrest During Pregnancy.” A second search was deemed necessary when the concept of “Education and Training” was added to the first search as a fourth concept and too many suitable articles were excluded. The Second Search consisted of two concepts: “Cardiovascular Pregnancy Complications” and “Education/Guidance.” Again, MeSH were utilized, as well as keywords and their related synonyms. The second search was also developed for the OVID Medline database. Once the search strategy was complete, it was submitted for approval and editing. The same publication date range was utilized when the search was run. Once each search was approved, they were both translated to two additional databases: Embase and The Cochrane Library. Final searches were run on November 29, 2018. An internal and external deduplication process was performed using the EndNote citation manager system. A manual deduplication search was performed on November 30, 2018. After deduplication the results from both searches were combined and deduplicated again.

In February 2019, the researchers decided to include the CINAHL Plus w/ Full Text database into the initial search to make sure all relevant articles were captured. It was also decided to update the search results from the first search initially run on November 2018. The search strategy was translated to the CINAHL database on February 7, 2019. The CINAHL search totals were combined with the initial search results from the searches (Medline OVID, Embase, and the Cochrane Library) run on November 29, 2018. A deduplication process was once again performed for the combined totals. The updates of the initial searches were also run on Medline OVID, Embase, and the Cochrane Library on February 7, 2019. The updates from the initial searches were combined and underwent the deduplication process on February 11, 2019. The updated search totals were added to the CINAHL search results and deduplicated again. The total 2,249 results of the updates and the CINAHL searches were then delivered to the researchers for review and to undergo the inclusion and exclusion process. The search strategies, in their entirety, are available in Appendix C.

The researchers reviewed the articles for relevance to the project. After categorization and determination of relevance, key questions were developed. The researchers abstracted and graded the literature and defined inclusion and exclusion criteria. Four independent reviewers selected studies to be included based on consensus on whether they met the inclusion and exclusion criteria. An independent reviewer was available for consultation if consensus was not reached among the reviewers but was not utilized. A synthesis of the relevant literature for each key question was performed focusing on validity and reliability of admissible evidence at the level of the individual study. The researchers developed overall summary statements regarding specific tasks for managing maternal cardiac arrest and extracted literature into a support summary table for each key question.
